# Bimetallic metal organic framework modified screen printed electrode for simultaneous voltammetric determination of calcium folinate and methotrexate

**DOI:** 10.5599/admet.3487

**Published:** 2026-08-08

**Authors:** Ameer Mahmood Shaker, Batool Nassir Hamran, Ala’a R. Shaker, Hussein Ali Al-Bahrani

**Affiliations:** 1 Ibn Sina University of Medical and Pharmaceutical Sciences, Baghdad Iraq; 2 Department of Basic Sciences, College of Dentistry, University of Kerbala, 56001, Karbala, Iraq; 3 Department of Chemistry, College of Science, Al-Nahrain University, Baghdad, Iraq; 4 College of Nursing, University of Al-Ameed, Department of Chemistry, College of Education for Pure Science, University of Kerbala, Iraq

**Keywords:** Cancer disease, chemotherapy drugs, electrochemical sensor

## Abstract

**Background and purpose:**

Calcium folinate (CFT) is a medication used as an antidote to methotrexate (MTT) and as a vitamin against anaemia. MTT is used to treat a variety of carcinomas, including acute lymphoblastic leukaemia, head and neck cancer, gastric cancer, breast cancer and choriocarcinoma.

**Experimental approach:**

A nanocomposite made of Fe and Mg linked to a 1,4-benzene dicarboxylate ligand metal organic framework (FeMg-BDC MOF) was created for this investigation. Using field emission scanning electron microscopy, the morphology and structure of the Mg-BDC MOF were examined. Next, a screen-printed electrode modified with a synthetic nanocomposite (FeMg-BDC MOF/SPE) was used as the working electrode for voltammetric detection of CFT.

**Key results:**

The FeMg-BDC MOF/SPE demonstrated high electrocatalytic activity for CFT oxidation as compared to unmodified SPE. The limit of detection is 0.04 μM, and under ideal conditions, the oxidation peak currents of CFT show a linear relationship with its concentration in the range of 0.1 to 390.0 μM. Additionally, when MTT was present, the FeMg-BDC MOF/SPE demonstrated good activity toward CFT determination. The separation of the oxidation peak potentials (255 mV) indicates that differential pulse voltammetry can be used to detect these two medications simultaneously.

**Conclusion:**

The devised sensor's applicability was confirmed with outstanding results using CFT and MTT assays in pharmaceutical specimens and urine samples.

## Introduction

Metal ions, together with organic ligands, form metal-organic frameworks (MOFs), a special family of three-dimensional crystalline materials [1]. The properties of coordination compounds depend on what kind of metal ions are used, as well as which bonding ligands are used [1]. Often, metal ions are linked through aromatic carboxylate-type ligands such as 1,4-benzene-dicarboxylate (BDC) [2], benzene-1,3,5-tricarboxylate (BTC) [3], while nitrogen heterocyclic-based ligands are also common, like pyrrole-, imidazole-, pyrazole-, triazole-, thiophene-, and furan-carboxylates [4].

MOFs have attracted considerable attention for their exceptional properties: crystallinity, high surface area, low density, excellent thermal stability, and tuneable chemical functionality [5]. Applications for MOFs are many and include gas storage and separation [6], sensors [7], drug release [8], lithium-ion batteries [9], and more.

Some conductive metal-based MOFs maintain a high density of active sites in electrochemical sensing, enabling high rates of electron transport across the electrolyte [8, 9]. To improve the sensitivity of detecting specific biological species in various fluids, it is desirable to design unique MOF-based sensors with a large surface area and high conductivity [10]. Notably, Mg-based MOFs have been utilized in several applications lately, including sensors [10].

The ionic radius and elastic coordinative habits of magnesium ions are said to be almost the same as those of other transition metal ions, like zinc and cobalt ions. Magnesium is also a promising metallic component in MOF synthesis, mainly because it is readily available, has low toxicity, and has a smaller environmental footprint [11]. The stronger links arising from the low electronegativity of Mg ions and the lone pairs on carboxyl groups are often described as strong binding interactions between the Mg ions and the carboxyl moieties. Through those interactions, 3D Mg-based MOFs can form, directly boosting gas adsorption, catalytic activity, sensor response, and energy-related applications [11].

Doctors prescribe methotrexate (MTT) as an anticancer medication. Chemically known as 2,4-diamino-N--10-methyl folic acid, MTT is used to treat a variety of carcinomas, including acute lymphoblastic leukaemia, head and neck cancer, gastric cancer, breast cancer, choriocarcinoma, and more [12]. MTT is absorbed into the body approximately one to two hours after oral intake.

MTT's therapeutic value is limited because it is highly toxic and inhibits the growth of healthy cells [13]. Therefore, to prevent the adverse consequences associated with MTT, there is a need for rapid, sensitive methods for its early, or at least timely, detection. Also, because MTT is not safe for long, it is crucial to monitor its therapeutic levels in the human body [13]. Many analytical options, such as enzyme multiplication immunoassay [14], radioimmunoassay [15], enzyme inhibition and protein binding [16], fluorimeter-based tests, microbiological methods [17], and high-performance liquid chromatography (HPLC) [18], have been introduced to track MTT. HPLC is the most widely used and reliable method for measuring MTT in blood samples [18]. These techniques are limited by their high cost and complexity. In addition to these traditional techniques, electrochemical techniques have been developed for MTT analysis [19]. Electroanalytical techniques are inexpensive, straightforward, sensitive, selective, quick to react, and simple to use for on-the-spot tests. Highly active electrocatalytic materials capable of selectively measuring MTT in both in vitro and real-sample settings are needed for these techniques [20].

Leucovorin calcium, another name for calcium folinate (CFT), is a folic acid derivative. This molecule is a naturally occurring substance found in living cells, where it is essential to several metabolic processes [21]. In the pharmaceutical industry, CFT is a medication used as an antidote to MTT and as a vitamin against anaemia [22]. For many years, it has been used in clinical settings as a rescue agent after MTT treatment [23]. CFT can be used to reduce MTT-induced toxicity without reducing its anticancer efficacy [24]. For the simultaneous measurement of MTT and CFT in biological samples, quick and straightforward analytical techniques are therefore used [25].

Since these methods are expected to be accurate and sensitive, and to detect and quantify different materials with their varying attributes in real-life samples, analytical chemists often face the challenge of developing approaches that enable rapid in situ analyses [25]. And for in situ, point-of-care setups aimed at quality control, environmental monitoring, and healthcare monitoring, people tend to rely less on current commercial lab tests. That’s mostly because they’re described as pretty complicated and also expensive [25]. Over the years, sensors built on screen-printed electrodes, or SPEs, have been among the key topics in electrochemical studies, mainly for fast, focused, portable, sensitive, inexpensive, and reliable assessments, plus they are claimed to open up new use cases [25]. The idea that screen printing can be much less expensive than traditional manufacturing routes was an important reason behind these big breakthroughs [26]. Screen printing has been suggested for the mass production of repeatable, affordable, dependable, single-use sensors for on-site monitoring in the microelectronics sector over the past 30 years [26]. On the one hand, SPEs enable the combination of functionalized compounds to produce many carbon electrodes in repeatable, inexpensive, and disposable formats. Well, SPEs get used in a bunch of areas of electrochemistry, especially for converting energy into usable forms and storing it, for detecting chemical or biological molecules, and even in microelectronics. On top of that, flexible electronics, a rapidly growing field in which you print electrical devices directly onto flexible plastic substrates such as polycarbonate, polyamide, and polyether ether ketone, often rely on these electrodes. When it comes to the most practical carbon types to deposit on them, people commonly choose graphite, activated carbon, or carbon black [27].

SPEs offer benefits over older electrode manufacturing methods because electrode thickness, surface characteristics, and overall composition can be adjusted relatively easily, and catalysts can be incorporated into the printing ink in a straightforward way. Plus, they allow more experimental statistical confirmation of the results, even if you end up having duplicate electrodes. The main downside is that they’re basically limited to flatbeds [28]. SPEs enable a range of tests using only small amounts of materials and reagents, without the need for electrode storage or pretreatment. In industries such as agriculture, pharmacy, medicine, the food sector, and the environment, these electrodes are frequently used for analysis [29,30].

To the best of our knowledge, most of the earlier published electrochemical studies relied on screen-printed or other electrode formats, and the electrodes were modified to determine CFT or MTT individually. So, in this paper, we report on how to craft a new screen-printed electrode (SPE) based on an Fe and Mg linked to a 1,4-benzene dicarboxylate ligand metal organic frameworks (FeMg-BDC MOF/SPE) and then evaluate its performance for the electrocatalytic detection of CFT in water-based solutions. We also evaluate the analytical performance of the modified electrode for CFT quantification in the presence of MTT.

## Experimental

### Apparatus and chemicals

An Autolab potentiostat/galvanostat equipped with General Purpose Electrochemical System (GPES) software (PGSTAT-302 N, Eco Chemie, The Netherlands) was used to run the electrochemical experiments. A silver pseudo-reference electrode and graphite working and auxiliary electrodes were used in the SPEs (Dropsens, DRP 110). Each solution was prepared freshly using double-distilled water. Merck (Darmstadt, Germany) supplied analytical-grade CFT, MTT, and all other reagents. Orthophosphoric acid, along with its salts, was used to prepare buffer solutions (PBS) over the pH range of 2.0 to 11.0.

### Synthesis of Fe and Mg linked to a 1,4-benzene dicarboxylate ligand metal organic frameworks

**Figure 1.**
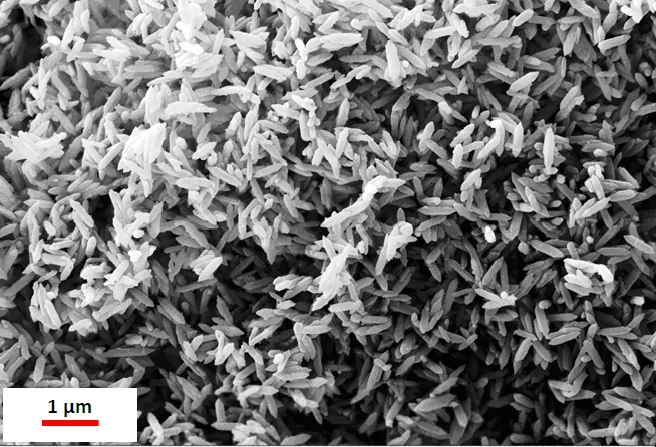
FE-SEM images of FeMg-BDC MOFs

At first, 2 mmol of 1,4-benzenedicarboxylic acid, also called BDC, and 2 mmol of a mix of metal salts FeCl_3_·6H_2_O together with Mg(NO_3_)_2_·6H_2_O, where the Fe^3+^ to Mg^2+^ molar ratio was set to 1/1, were poured into 20 mL of DMF. The whole thing was kept in constant agitation until a fairly uniform solution came together. Then, 4 mL of a 0.2 M NaOH solution was added, and the suspension was stirred for about 20 min before being transferred to an oven at 100 °C for 12 hours. Afterward, it was taken out and left to cool down to room temperature. The resulting MOFs were then dried at 80 °C for 18 hours and, in between, were repeatedly centrifuged in ethanol to remove residual or unreacted materials. Figure 1 shows the field emission scanning electron microscopy (FE-SEM) images of FeMg-BDC MOFs.

### Preparation of screen-printed electrode modified with Fe and Mg linked to a 1,4-benzene dicarboxylate ligand metal organic frameworks

1.0 mg of FeMg-BDC MOFs heterostructures were ultrasonically dispersed in 1.0 mL of deionized water for 15 minutes at room temperature to provide a homogeneous suspension for the modified electrode manufacturing (FeMg-BDC MOFs/SPE). To create FeMg-BDC MOF/SPE, 3.0 μL of FeMg-BDC heterostructures suspension was drop-cast onto the SPCE surface and allowed to dry at room temperature.

### Procedure of real sample preparation

#### Preparation of methotrexate injection

At first, a 100 mL volumetric flask was filled with 1 mL of the MTT injection (the vial containing 25 mg mL^-1^ injectable solution) and then brought up to the mark with PBS 0.1 M (pH 7.0). After that, a 25 mL volumetric flask was filled to the top with PBS 0.1 M (pH 7.0), but only after a selected volume of the freshly prepared solution was transferred into it. The mixture was subsequently placed into the electrochemical setup, kind of as a true specimen.

For the estimation of those species, the standard addition approach was used: the unspiked injection sample and spiked injection samples prepared with standard MTT and CFT solutions at different concentrations, were introduced into the electrochemical cell. Afterward, the differential pulse voltammetry (DPV) response was recorded. Each concentration was basically repeated five times, not once.

#### Preparation of calcium folinate injection

First, about 100 mL of a volumetric flask was prepared by adding 1 mL of the CFT injection, which came from a vial containing a 10 mg mL^-1^ injection solution. Then it was diluted until the graduation mark, using PBS 0.1 M (pH 7.0). After that, a 25 mL volumetric flask was filled to the top with PBS 0.1 M (pH 7.0), but only after a specific volume of the prepared solution was poured into it. This final mixture was then loaded into an electrochemical device as the real sample for analysis. To quantify these analytes using the standard addition approach, unspiked and spiked injection specimens were prepared. Each spiked set included standard solutions of CFT and MTT at several concentrations, and all were transferred into the electrochemical cell. The corresponding differential pulse voltammograms (DPVs) were collected and saved. For every concentration level, the whole measurement was repeated 5 times.

#### Urine analysis

Ten millilitres of urine samples were also centrifuged at 2000 rpm for fifteen minutes. A portion of the resulting supernatant was transferred to a 25 mL volumetric flask and, after passing through a 0.45 μm filter, diluted with PBS (pH 7.0) to the appropriate volume. Afterward, varying amounts of CFT and MTT were added to the diluted urine samples. Lastly, the conventional addition procedure was used to quantify the concentrations of CFT and MTT.

## Result and discussions

### Oxidation of calcium folinate at Fe and Mg linked to a 1,4-benzene dicarboxylate ligand metal organic frameworks/screen printed electrode

The pH of the aqueous solution affects how CFT behaves electrochemically and it appears that achieving oxidation of CFT requires adjusting the solution pH. Therefore, cyclic voltammetry was used to examine the electrochemical behaviour of CFT on the surface of the FeMg-BDC MOF/SPE in 0.1 M phosphate buffer solutions at various pH values (4.0< pH <9.0). It was found that neutral conditions were more favourable for the oxidation of CFT on the surface of the FeMg-BDC MOF/SPE than acidic or basic media. The cyclic voltammogram of CFT shows this as a progressive increase in the anodic peak current. Thus, pH 7.0 was chosen as the optimum pH for the electrocatalytic oxidation of CFT on the surface of the FeMg-BDC MOF/SPE.

**Figure 2.**
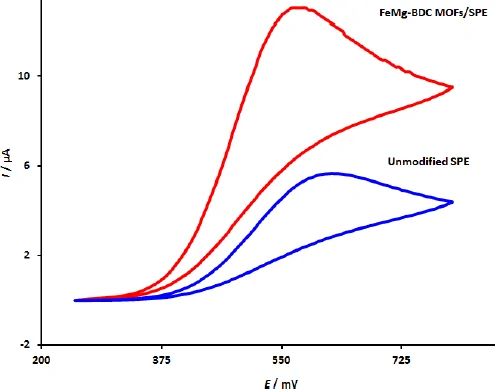
CVs of unmodified SPE and FeMg-BDC MOF-modified SPE at a scan rate of 50 mV s^-1^ in a 0.1 M phosphate buffer solution containing 100.0 μM CFT (pH 7.0)

The cyclic voltammetry (CV) responses for the electrochemical oxidation of 100.0 μM CFT at unmodified SPE and FeMg-BDC MOF/SPE are shown in Figure 2.

As shown, the corresponding current peak potential at FeMg-BDC MOFs/SPE is around 570 mV, whereas the anodic peak potential for CFT oxidation at the unmodified SPE is 640 mV. These findings show that, in comparison to unmodified SPE, the peak potential for CFT oxidation at the FeMg-BDC MOF/SPE shifts by 70 mV toward negative values. The findings show that the electrode's performance against CFT oxidation has been greatly enhanced by FeMg-BDC MOF.

**Figure 3.**
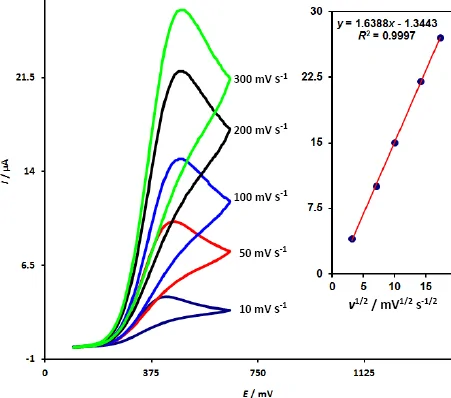
Cyclic voltammograms of FeMg-BDC MOF/SPE in 0.1 M phosphate buffer solution (pH 7.0) with 80.0 μM CFT at different scan rates. The variation of anodic peak current *vs. v*^1/2^ is shown in the insets

Using cyclic voltammetry, the impact of scan rate on the oxidation of CFT at the FeMg-BDC MOF/SPE was examined (Figure 3). The oxidation peak potential shifted to more positive values with increasing scan rate, as shown in Figure 3, indicating kinetic hindrances in the electrochemical process. Additionally, it was discovered that a plot of peak height (*I*_p_) *vs.* the square root of scan rate (*v*^1/2^) was linear in the region of 10 to 300 mV s^-1^ (Inset of Figure 3), indicating that the process is diffusion rather than surface-controlled at high enough overpotential.

### Chronoamperometric measurements

**Figure 4.**
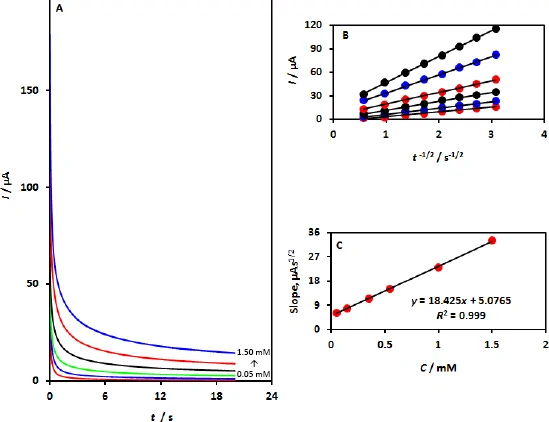
(A) Chronoamperograms for various CFT concentrations (0.05, 0.15, 0.35, 0.55,1.0 and 1.50 mM) obtained at FeMg-BDC MOF/SPE in a 0.1 M phosphate buffer solution (pH 7.0). (B) *I vs. t*^-1/2^ plots derived from chronoamperograms. (C) Plot of the slope of the straight lines against the concentration of CFT

Chronoamperometric measurements were performed by setting the FeMg-BDC MOF/SPE working electrode potential at 0.6 V for the different concentrations of CFT in PBS (pH 7.0). The current-time curves are shown in Figure 4A. The Cottrell equation describes the current for the electrochemical reaction under the mass transport-constrained condition for an electroactive material (in this example, CFT) with a diffusion coefficient of *D*. The best fits for various CFT concentrations were found using experimental plots of *I vs. t*^-1/2^ (Figure 4B). Plotting the slopes of the obtained straight lines against CFT concentration was the next step (Figure 4C). The mean value of the *D* was determined to be 2.98×10^-5^ cm^2^ s^-1^ using the Cottrell equation and the resultant slope.

### Calibration plot and limit of detection

**Figure 5.**
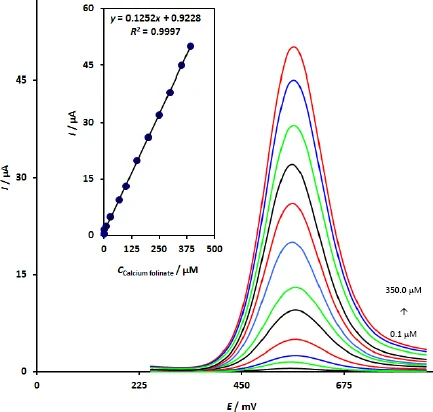
FeMg-BDC MOF/SPE differential pulse voltammograms in a 0.1 M phosphate buffer solution (pH 7.0) with varying CFT concentrations. 0.1, 2.5, 10.0, 30.0, 70.0, 100.0, 150.0, 200.0, 250.0, 300.0, 350.0 and 390.0 μM of CFT. Plots showing the electrocatalytic peak current as a function of CFT concentration in the range of 0.1 to 39.0 μM are displayed in the inset

The concentration of CFT was calculated from the DPV measurements (Figure 5). A current peak height varies linearly with concentration, and a linear segment with a slope of 0.1252 μA μM^-1^ in the concentration range of 0.1 to 390.0 μM was obtained. It was discovered that the CFT detection limit, 3*σ* (*σ* - standard deviation), was 0.04 μM.

### Simultaneous determination of calcium folinate and methotrexate

**Figure 6.**
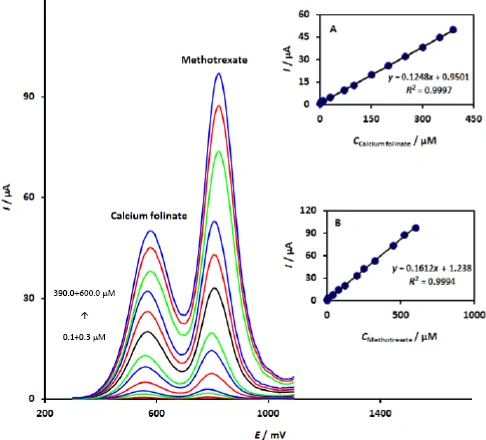
DPVs of FeMg-BDC MOF/SPE in a 0.1 M phosphate buffer solution (pH 7.0) with varying CFT+MTT concentrations in μM, from inner to outer: 0.1+0.3, 2.50+1.0, 10.0+7.5, 30.0+40.0, 70.0+80.0, 100.0+120.0, 150.0+200.0, 200.0+250.0, 250.0+325.0, 300.0+450.0, 350.0+525.0 and 390.0+600.0 μM. Plots of *I*_p_ *vs.* CFT and MTT concentrations are shown in insets (A) and (B)

As far as we are aware, no study has been published on the simultaneous measurement of CFT and MTT utilizing FeMg-BDC MOF/SPE. Thus, the primary goal of this study was to use FeMg-BDC MOF/SPE to concurrently detect CFT and MTT. This was accomplished by recording the DPVs while concurrently altering the CFT and MTT concentrations. The voltammetric data revealed distinct anodic peaks at 570 and 825 mV, corresponding to the oxidation of CFT and MTT, respectively. This suggests that it is possible to simultaneously determine these chemicals using the FeMg-BDC MOF/SPE, as shown in Figure 6.

It was discovered that the modified electrode's sensitivity to CFT oxidation was 0.1252 μA μM^-1^. This is very similar to the value obtained without MTT (0.1248 μA μM^-1^), suggesting that the oxidation processes of these compounds at the FeMg-BDC MOF/SPE are independent, enabling simultaneous determination of their mixtures without significant interference.

### Real sample analysis

#### Determination of calcium folinate and methotrexate in pharmaceutical preparations

The suggested approach was also used to figure out CFT and MTT in CFT and MTT injections, in order to check how well it really works analytically, or at least that’s how it was framed. Measurements were made to estimate the amounts of CFT and MTT in the pharmaceutical preparations using repeated differential pulse voltammetric readings (*n* = 5) obtained from diluted analytes and from samples spiked with known quantities of CFT and MTT. Table 1 shows the outcomes, more or less. Then, by comparing these results with the information on the medicinal product labels, the trustworthiness of the suggested modified electrode was also evaluated (Table 2). The relative standard deviations (RSD, %) and recovery rates for the spiked samples appear acceptable, as shown in Table 1. Also, in Table 2, the statistics indicate that the results obtained by using FeMg-BDC MOF/SPE line up quite well with the values stated on the labels. Overall, this modified electrode can be used effectively to determine MTT and CFT, either separately or simultaneously, in pharmaceutical formulations.

**Table 1.** The application of FeMg-BDC MOF/SPE for simultaneous determination of CFT and MTT (*n* = 5)

Sample	Amount, μM				Recovery, %		RSD, %	
	Spiked		Found					
	CFT	MTT	CFT	MTT	CFT	MTT	CFT	MTT
CFT injection	0	0	2.9	-	-	-	3.3	-
	2.0	5.0	4.8	5.1	97.9	102.0	2.3	3.2
	3.0	6.0	6.1	5.8	103.4	96.7	3.0	1.8
	4.0	7.0	6.8	7.2	98.5	102.9	1.9	2.4
	5.0	8.0	8.0	7.9	101.3	98.7	2.5	2.6
MTT injection	0	0	-	4.1	-	-	-	2.8
	5.5	1.0	5.4	5.2	98.2	102.0	3.6	2.7
	6.5	2.0	6.7	5.9	103.1	96.7	2.5	1.9
	7.5	3.0	7.4	7.2	98.7	101.4	2.0	2.6
	8.5	4.0	8.6	8.0	101.2	98.7	2.3	2.4

**Table 2.** Comparison of the total values of CFT and MTT of some pharmaceutical preparations obtained using FeMg-BDC MOF/SPE with declared values in the label of the samples (*n* = 5)

Samples	Declared concentration, mg mL^-1^	Found concentration, mg mL^-1^	RSD, %
CFT injection	10.00	10.03	2.6
MTT injection	25.00	24.92	2.5

#### Determination of calcium folinate and methotrexate in urine sample

The suggested approach was also used to determine CFT and MTT in a urine sample to assess the analytical utility of the method. Table 3 shows the results obtained when identifying the two species in the urine sample. For both CFT and MTT, a reasonable recovery of the experimental results was seen. The mean relative standard deviation R.S.D. suggested the method was repeatable.

**Table 3.** The application of FeMg-BDC MOF/SPE for simultaneous determination of CFT and MTT in a urine sample (*n* = 5)

Amount, μM				Recovery, %		RSD, %	
Spiked		Found					
CFT	MTT	CFT	MTT	CFT	MTT	CFT	MTT
0	0	-	-	-	-	-	-
4.0	4.5	4.1	4.4	102.5	97.8	2.5	3.6
6.0	6.5	5.9	6.6	98.3	101.5	3.5	2.7
8.0	8.5	8.1	8.4	98.9	98.9	1.9	2.8
10.0	10.5	9.9	10.9	103.8	103.8	2.0	2.2

## Conclusions

The current work employed a screen-printed electrode modified with FeMg-BDC MOF to measure CFT in the presence of MTT. The CV and DPV analyses demonstrated the modified electrode's efficient electrocatalytic activity in reducing the anodic overpotential for CFT oxidation and fully resolving its anodic wave from MTT. CFT and MTT may be determined simultaneously in mixtures with minimal interference, owing to the measured potential difference of 180 mV between them.
